# Autosomal dominant osteopetrosis associated with renal tubular acidosis is due to a *CLCN7* mutation

**DOI:** 10.1002/ajmg.a.37755

**Published:** 2016-08-19

**Authors:** Sian E. Piret, Caroline M. Gorvin, Anne Trinh, John Taylor, Stefano Lise, Jenny C. Taylor, Peter R. Ebeling, Rajesh V. Thakker

**Affiliations:** ^1^ Academic Endocrine Unit Oxford Centre for DiabetesEndocrinology and MetabolismChurchill HospitalUniversity of OxfordOxfordUnited Kingdom; ^2^ Faculty of Medicine Nursing and Health SciencesDepartment of MedicineSchool of Clinical SciencesMonash Medical CentreMonash UniversityClaytonVictoriaAustralia; ^3^ Oxford Medical Genetics LaboratoriesChurchill HospitalOxford University Hospitals NHS TrustOxfordUnited Kingdom; ^4^ Wellcome Trust Centre for Human GeneticsOxfordUnited Kingdom; ^5^ Oxford NIHR Comprehensive Biomedical Research CentreOxfordUnited Kingdom

**Keywords:** exome sequencing, bone, chloride‐channel, Albers–Schonberg disease

## Abstract

The aim of this study was to identify the causative mutation in a family with an unusual presentation of autosomal dominant osteopetrosis (OPT), proximal renal tubular acidosis (RTA), renal stones, epilepsy, and blindness, a combination of features not previously reported. We undertook exome sequencing of one affected and one unaffected family member, followed by targeted analysis of known candidate genes to identify the causative mutation. This identified a missense mutation (c.643G>A; p.Gly215Arg) in the gene encoding the chloride/proton antiporter 7 (gene *CLCN7*, protein CLC‐7), which was confirmed by amplification refractory mutation system (ARMS)‐PCR, and to be present in the three available patients. CLC‐7 mutations are known to cause autosomal dominant OPT type 2, also called Albers–Schonberg disease, which is characterized by osteosclerosis, predominantly of the spine, pelvis and skull base, resulting in bone fragility and fractures. Albers–Schonberg disease is not reported to be associated with RTA, but autosomal recessive OPT type 3 (OPTB3) with RTA is associated with carbonic anhydrase type 2 (*CA2*) mutations. No mutations were detected in *CA2* or any other genes known to cause proximal RTA. Neither *CLCN7* nor *CA2* mutations have previously been reported to be associated with renal stones or epilepsy. Thus, we identified a *CLCN7* mutation in a family with autosomal dominant osteopetrosis, RTA, renal stones, epilepsy, and blindness. © 2016 The Authors. *American Journal of Medical Genetics Part A* Published by Wiley Periodicals, Inc.

## INTRODUCTION

Osteopetrosis (OPT) comprises a heterogeneous group of diseases characterized by high bone density with increased cortical and trabecular bone thickness and susceptibility to fractures, that may be due either to a reduction in the number of differentiated osteoclasts, or to a defect in osteoclast function, resulting in reduced bone resorption [Aggarwal, 2013]. The clinical severity of these features is variable and OPT forms have traditionally been distinguished by their age of onset, the affected bones, other associated clinical features such as renal tubular acidosis, neurological impairment, retinal atrophy, or immune defects [Bollerslev et al., 2013; Sobacchi et al., 2013], and heritability, with autosomal dominant OPT (ADO) having a later and less severe onset than autosomal recessive OPT (ARO). However, there are clinical overlaps between these forms of OPT, such that an intermediate ARO (IARO) form has also been identified, and there are also overlaps with other high bone density disorders which may have similar radiological and clinical features [Warman et al., 2011; Aggarwal, 2013], making it difficult to establish a diagnosis on the basis of clinical features alone. For example, ADO Type 1 (OPTA1), due to mutations of low density lipoprotein receptor‐related protein 5 (LRP5), has been reclassified as a high bone mass disease rather than classical OPT. To date, defects in 25 different genes have been demonstrated to cause OPT or other high bone density disorders (Supplementary Table SI), and different mutations in the chloride/proton antiporter 7 (gene symbol *CLCN7*, and protein symbol CLC‐7) have been reported to cause ADO, ARO, or IARO [Bollerslev et al., 2013; Sobacchi et al., 2013]. Moreover, mutations of CLC‐7, which provides a Cl^−^ countercurrent to allow acidification of the osteoclast resorption lacuna [Bollerslev et al., 2013], cause ADO type 2 (OPTA2), also called Albers–Schonberg disease, which is characterized by osteosclerosis predominantly affecting the spine, pelvis and skull base and resulting in bone fragility, fractures, and dental abscesses [Bollerslev et al., 2013].

**Figure 1:**
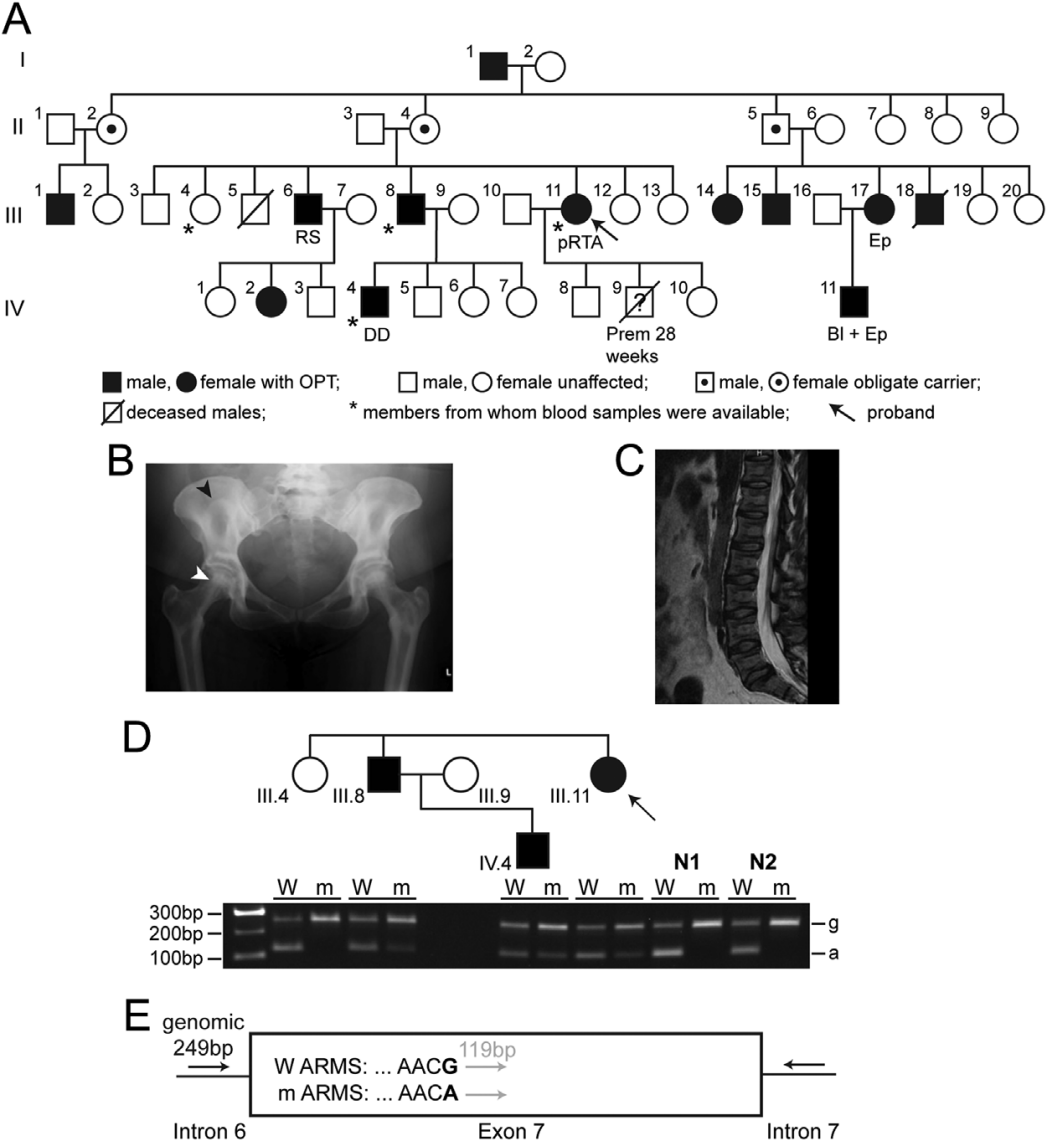
**A:** Pedigree of proband (arrowed) with OPT and pRTA. Inheritance of OPT by a son (individual IV.4), with developmental delay (DD), from his father (III.8) demonstrates male‐to‐male transmission, a hallmark of an autosomal dominant disorder. Individual III.6, at age 24 years had back pain, and X‐rays revealed dense bones, consistent with OPT; he has not had any fractures but has multiple renal stones (RS) containing calcium. His daughter (IV.2) had fractured toes and radiographs revealed dense bones, consistent with OPT. A maternal cousin (III.17) had multiple humeral and femoral fractures, and was diagnosed with OPT aged 38 years; she has epilepsy (Ep) with seizures occurring 8–10 times per day despite medical therapy. Her son (IV.11), born blind (Bl), also has epilepsy, is now aged 35 years, and has suffered from multiple fractures due to OPT. The mother (II.4) and father (II.3) of the proband have not developed any clinical or radiological signs of OPT. None of the relatives of the proband with OPT and pRTA, has hyperchloremic metabolic acidosis. Prem: premature birth. **B:** Pelvic radiograph of proband, showing “bone within bone” appearance of sacral ala and femoral heads (arrowheads). **C:** Lateral MRI scan of proband, showing a rugger‐jersey spine. **D:** *CLCN7* c.643G>A mutation, confirmed by ARMS‐PCR in the proband, and shown to be present in affected relatives, and absent in an unaffected relative, and in 100 unrelated unaffected individuals, of whom 2 (N1 and N2) are shown. ARMS‐PCR products (wild‐type [W] and mutant [m]) are shown below each individual; with “g” and “a” denoting genomic and ARMS bands, respectively. pRTA is likely due to the *CLCN7* mutation, rather than a second mutation in a gene known to cause pRTA, as ∼88% of the coding regions of genes were covered by exome sequencing at a read depth that would allow detection of such variants. **E:** Location of primers in relation to exon 7 and the mutated nucleotide (bold).

Here, we report a family with ADO with variable expressivity, associated with renal tubular acidosis (RTA), renal stones, developmental delay, blindness and epilepsy (Fig. 1A), a combination of features not previously reported. RTA is not a feature of OPTA2, but is only associated with ARO type 3 (OPTB3), due to mutations of the gene encoding carbonic anhydrase type II (gene symbol *CA2*, protein symbol CAII) [Sobacchi et al., 2013]. RTA, which may be of proximal (pRTA) or distal (dRTA) tubular origin, occurring without OPT may be associated with mutations in >10 genes (Supplementary Table SII). Thus, >35 genes may be associated with OPT (Supplementary Table SI) and pRTA (Supplementary Table SII), and to determine the causative mutation in this unusual family, we undertook exome sequencing and cosegregation analysis.

## MATERIALS AND METHODS

### Patients and Clinical Findings

Informed consent and venous blood samples were obtained from the four available individuals (three affected, one unaffected) of the family with ADO (Fig. 1A), using protocols approved by the Multicentre Research Ethics Committee (UK) (MREC/02/2/93), and local and national ethics committees (Australia). The proband (Fig. 1A, individual III.11), a 49‐year‐old woman, had presented at the age of 2 years with a fractured tibia caused by falling out of bed. She had fractures of the opposite tibia aged 3 years, and ankle, aged 13 years, that required open reduction, and internal fixation. Radiographs revealed increased bone density, consistent with OPT. She continued to have fractures of her humeri, femur, tibiae, fingers and toes, such that she became wheelchair bound by 35 years of age. At age 36 years, a dual‐energy X‐ray absorptiometry (DXA) scan revealed a Z‐score of +5.6 at the lumbar spine and +3.5 at the proximal hip, and concurrent X‐rays demonstrated increased bone density with a “bone within bone” appearance of the sacral ala and both femoral heads (Fig. 1B), a typical feature of OPT. MRI has demonstrated a rugger‐jersey spine (Fig. 1C), which is associated with OPTA2, although she does not have osteosclerosis of the skull. Serum concentrations of C‐telopeptide (CTX) of 85 ng/L (reference range 100–700 ng/L) and N‐terminal propeptide of type one collagen (P1NP) of 9 μg/L (reference range < 59μg/L) are consistent with a low bone turnover state, but serum lactate dehydrogenase (LDH) isoenzymes were elevated at 533 U/L (reference range 210–420U/L), consistent with OPTA2 [Whyte et al., 2010]. In her 4th decade, she had severe asthma episodes that required high‐dose glucocorticoids, and despite chronic carbon dioxide retention and a respiratory acidosis, her plasma bicarbonate levels were inappropriately normal and associated with significant hypokalemia, consistent with a pRTA. However, she did not have phosphaturia, uricosuria, glycosuria or aminoaciduria, thereby excluding a renal Fanconi syndrome, and she was able to acidify her urine when challenged with an ammonium chloride loading test from pH > 5.5 to pH < 5.3, thereby excluding a dRTA (Supplementary Table SIII). In addition, her plasma bicarbonate remained at the lower end of the normal range (22.1–24.6 mmol/L), despite oral bicarbonate treatment, whilst her urinary pH rose to 6.55–6.92, consistent with a defect in renal bicarbonate reabsorption, and a pRTA. However, she did not have intracerebral calcifications, cognitive impairment, and hearing loss, which occur in patients with OPTB3, who have autosomal recessive OPT with pRTA. Moreover, OPT in the proband's family was inherited as an autosomal dominant trait (Fig. 1A) in association with pRTA, blindness, epilepsy and renal stones, a combination that has not previously been reported.

### Exome Sequencing and Variant Confirmation

Leukocyte DNA was extracted and used for exome capture, utilizing DNA from one affected and one unaffected individual. Variants were confirmed by DNA Sanger sequence analysis, and amplification‐refractory mutation system (ARMS)‐PCR (Supplementary Methods).

## RESULTS

Exome sequencing identified a single nucleotide variant, c.643G>A, in *CLCN7*, encoding a missense substitution, Gly215Arg, which has been reported to be a mutation causing OPT in multiple families with OPT2A [Cleiren et al., 2001; Bollerslev et al., 2013]. This variant, located in exon seven of *CLCN7*, was confirmed by DNA Sanger sequence analysis (data not shown) in the proband and by ARMS‐PCR to occur as a heterozygous variant in the three affected individuals and to be absent in the unaffected relative and 100 control unaffected, and unrelated individuals of European origin (Fig. 1D, E). This *CLCN7* variant was also absent from >57,000 exomes from the Exome Aggregation Consortium (ExAC) database. Thus, these data demonstrate that this Gly215Arg CLC‐7 mutation is the cause for this autosomal dominant form of OPT. Samples from other family members were unavailable for further segregation analysis, and variants of *CA2* or other candidate genes for pRTA (Supplementary Table SII) were not identified.

## DISCUSSION

Our study reports a family with ADO in association with pRTA, renal stones, epilepsy, and blindness. Such a combination of clinical features has not been previously reported. The OPT was identified to be due to a CLC‐7 mutation, Gly215Arg, and thus, this family has Albers–Schonberg disease, which is characterized by diffuse, symmetrical osteosclerosis mainly affecting the spine, pelvis and skull, increased fracture rate, and severe vision loss beginning in childhood in ∼20% of patients [Waguespack et al., 2007; Bollerslev et al., 2013]. Moreover, ∼20–40% of CLC‐7 mutation carriers remain asymptomatic, consistent with variable expressivity [Waguespack et al., 2007; Bollerslev et al., 2013]. Thus, these features of Albers–Schonberg disease are consistent with those observed in this family (Fig. 1A), in which there are 12 individuals affected with ADO, with one having blindness, and there are three asymptomatic obligate heterozygotes, indicating variable expressivity. However, CLC‐7 mutations have not previously been reported in association with renal abnormalities, such as pRTA and renal stones, or with epilepsy, thereby indicating that Albers–Schonberg disease may be associated with additional clinical features.

CLC‐7 is a chloride/proton antiporter that is ubiquitously expressed, and found predominantly on the membranes of endolysosomal vesicles [Lange et al., 2006]. In osteoclasts, CLC‐7 is also found in the ruffled membrane, where with its β‐subunit, the OPT‐associated transmembrane protein 1 (OSTM1), mutations of which result in ARO type 5 (OPTB5) (Supplementary Table SI), it functions to provide a Cl^−^ countercurrent for acidifying the resorption lacuna [Lange et al., 2006]. The CLC‐7 Gly215Arg mutation reduces osteoclast activity by ∼80–90% [Henriksen et al., 2004; Schulz et al., 2010], and *Clcn7* null (*Clcn7* ^−/−^) mice develop severe OPT and retinal degeneration [Kornak et al., 2001], but are not reported to have RTA. However, renal proximal tubules (PT) from mice with PT‐specific deletion of *Clcn7* had enlarged endolysosomal vesicles in which degradation of endocytosed protein was impaired, suggesting an important role for CLC‐7 in the renal PT [Wartosch et al., 2009]. Moreover, rare variants, deletions, duplications or copy number variations were not detected in other pRTA causing genes in the family with OPT and pRTA, thereby indicating that pRTA may be a new clinical feature associated with CLC‐7 mutations, and studies of additional OPT patients harboring CLC‐7 mutations may help to further elucidate this.

The variable clinical features of OPT in the family (e.g., epilepsy in individuals III.17 and IV.11 [Fig. 1A]), is consistent with previous observations in families with ADO [Bollerslev et al., 2013], and may be due to digenic inheritance (co‐inheritance of two rare recessive disorders), or genetic modifiers. For example, the occurrence of OPT and dRTA in a Turkish kindred is due to digenic inheritance comprising: a homozygous deletion of *TCIRG1*, resulting in ARO type 1 (OPTB1) (Supplementary Table SI); and a homozygous mutation of the ATPase, proton (H^+^) transporting lysosomal 50/58 kDa VI subunit B1 (*ATP6V1B1*) resulting in dRTA [Borthwick et al., 2003]. Samples from individuals III.17 and IV.11 (Fig. 1A) with OPT and epilepsy were not available to investigate for such digenic inheritance of epilepsy and OPT, which was due to the *CLCN7* mutation. Genetic modifiers may also contribute to variable expressivity of OPT, as illustrated by breeding of a knock‐in mouse model with a CLC‐7 Gly213Arg mutation, the equivalent of the human CLC‐7 Gly215Arg mutation, onto five different genetic backgrounds, which revealed differences in the severity of bone disease, such as the bone volume/total volume, which was increased by ∼two fold but only ∼1.4 fold in heterozygous mutant mice on 129 and D2 backgrounds, respectively, when compared to wild‐type mice [Alam et al., 2014].

Thus, our identification of a CLC‐7 mutation in a family with OPT and multiple clinical features, reveals a potential role for CLC‐7 in renal proximal tubular physiology.

## WEB RESOURCES

Exome Aggregation Consortium (ExAC), http://exac.broadinstitute.org

## Supporting information

Additional supporting information may be found in the online version of this article at the publisher's web‐site.

Supporting Information.
