# Supplementary material for: Autosomal dominant osteopetrosis associated with renal tubular acidosis is due to a CLCN7 mutation
Source: Am J Med Genet A. 2016 Aug 19;170(11):2988–92. doi: 10.1002/ajmg.a.37755 (PMC5132132; doi:10.1002/ajmg.a.37755)
Supplement: Supplementary file 1 — Supporting Information. [file AJMG-170-2988-s001.docx]

**ONLINE SUPPLEMENTARY METHODS**

**Exome sequencing and data analysis**

Leukocyte DNA was extracted from venous blood using the Gentra Puregene blood kit (Qiagen) and standard protocols, quantified using the High Sensitivity Qubit system (Invitrogen), and assessed for integrity using an agarose gel, as described [Nesbit et al. 2013]. Exome capture was performed in one affected and one unaffected individual using the SeqCap EZ Human Exome Library v2.0 (Roche NimbleGen), and sequenced using a 100 bp paired-end read protocol on an Illumina HiSeq [Taylor et al. 2015]. Approximately 15 Gb of sequence were obtained for the affected individual, providing at least 10x vertical read depth over ~90% of the coding exome, as specified by the consensus coding sequence (CCDS) project. Reads were aligned to Human Genome version 19 (hg19) with Stampy (v1.0.20) [Lunter and Goodson 2011] and variant calling of single nucleotide variants (SNVs) and short insertions and deletions (indels) was undertaken using Platypus (v0.5.2) [Rimmer et al. 2014]. The analysis of coding variants was restricted to 36 genes associated with osteopetrosis or pRTA (Online Supplementary Tables 1 and 2), using the Illumina VariantStudio data analysis software. Variants with a minor allele frequency >5% within all populations of 1000 genomes data (April 2012 phase 1 call set (v3 update)) and from the National Heart, Lung, and Blood Institute (NHLBI) exome sequencing project (Exome Variant Server, NHLBI GO Exome Sequencing Project (ESP), Seattle, WA [November 2012 accessed]) were excluded. The pathogenicity of variants was inferred from several criteria: allele frequency within the NHLBI exome sequencing project and the Exome Aggregation Consortium (ExAC), Cambridge, MA [date accessed: October 2015], amino acid conservation, physicochemical alterations in amino acid substitutions, splice site prediction algorithms (NNSPLICE, MaxEntScan, GeneSplicer, and SpliceSiteFinder-Like) and literature review. Copy number variations (exonic deletions and duplications) were scrutinized using ExomeDepth [Plagnol et al. 2012].

**Sanger sequence analysis and amplification refractory mutation system (ARMS)-PCR**

Candidate variants were confirmed by DNA Sanger sequence analysis [Nesbit et al 2013] and cosegregation analysis was undertaken using ARMS-PCR [Williams et al. 2009] in independently obtained leukocyte DNA from the three affected and one unaffected family members, as previously described [Nesbit et al 2013; Williams et al 2009]. Variants were also shown to be absent in 100 unaffected, unrelated individuals of European origin by ARMS-PCR [Williams et al 2009].

**WEB RESOURCES**

The URLs for web resources are as follows:

Exome Variant Server, http://www.evs.gs.washington.edu/EVS;

Exome Aggregation Consortium (ExAC), http://exac.broadinstitute.org;

NNSPLICE, http://www.fruitfly.org/seq_tools/splice.html;

MaxEntScan, http://genes.mit.edu/burgelab/maxent/Xmaxentscan_scoreseq.html;

GeneSplicer, http://www.cbcb.umd.edu/software/GeneSplicer/gene_spl.shtml; and SpliceSiteFinder-Like http://www.interactive-biosoftware.com.

**ONLINE SUPPLEMENTARY RESULTS**

Exome sequencing resulted in read depths of ≥10 reads across ~88% of the coding regions of the known candidate genes for osteopetrosis and pRTA (Online Supplementary Tables I and II) in both the affected (Online Supplementary Table IV) and unaffected individuals. The candidate genes were then analyzed for variants that were present in the affected individual but absent in the unaffected individual. Variants reported at a minor allele frequency of >5% were excluded, which resulted in seven variant calls. Five of these were spurious variant calls that occurred after short mononucleotide tracts and were therefore excluded as sequence artifacts, and the remaining two variants were single nucleotide variants: c.28G>A in *SOST*, encoding a missense substitution, c.##X>Y p.(Val10Ile) in Sclerostin (SOST); and c.643G>A in *CLCN7*, encoding a missense substitution, p.(Gly215Arg). The *SOST* variant (rs17882143) has previously been reported to be benign [Uitterlinden et al. 2004], whereas the *CLCN7* variant has been reported to be a pathogenic mutation causing OPT in multiple families [Bollerslev et al. 2013; Cleiren et al. 2001]. No copy number variations were present in any of the candidate genes.

**ONLINE SUPPLEMENTARY TABLE I.** Candidate genes for osteopetrosis (OPT)

| Disorder | Symbol | OMIM # | Locus | Gene name | Protein | Number of exons (longest isoform)^a^ |
| --- | --- | --- | --- | --- | --- | --- |
| Autosomal dominant OPT & high bone mass | OPTA1 | 607634 | 11q13.4 | *LRP5* | Low density lipoprotein (LDL) receptor-related protein 5 | 23 (002335) |
|  | OPTA2 | 166600 | 16p13 | *CLCN7* | Chloride/proton antiporter 7 | 25 (001287) |
|  | BOS | 166700 | 12q14 | *LEMD3* | LAP2, emerin, MAN1 (LEM) domain-containing 3 | 13 (014319) |
|  | CMDD | 123000 | 5p15.1 | *ANKH* | ANKH inorganic pyro-phosphate transport regulator | 12 (054027) |
|  | CAEND | 131300 | 19q13.2 | *TGFB1* | Transforming growth factor, beta 1 | 7 (000660) |
|  | TDO | 190320 | 17q21 | *DLX3* | Distal-less homeobox 3 | 3 (005220) |
|  | CDD | 122860 | 17q21 | *SOST* | Sclerostin | 2 (025237) |
| Autosomal recessive OPT | OPTB1 | 259700 | 11q13 | *TCIRG1* | ATPase, H+ transporting, lysosomal V0 subunit A3 | 20 (006019) |
|  | OPTB2 | 259710 | 13q14.11 | *TNFSF11* | Tumour necrosis factor (ligand) superfamily, member 11 | 5 (003701) |
|  | OPTB3 | 259730 | 8q21.2 | *CA2* | Carbonic anhydrase type 2 | 7 (000067) |
|  | OPTB4 | 611490 | 16p13 | *CLCN7* | Chloride/proton antiporter 7 | 25 (001287) |
|  | OPTB5 | 259720 | 6q21 | *OSTM1* | OPT associated transmembrane protein | 6 (014028) |
|  | OPTB6 | 611497 | 17q21.3 | *PLEKHM1* | Pleckstrin homology domain-containing protein, family M, member 1 | 12 (014798) |
|  | OPTB7 | 612301 | 18q22.1 | *TNFRSF11A* | Tumour necrosis factor receptor superfamily, member 11a | 10 (003839) |
|  | OPTB8 | 615085 | 7p15.2 | *SNX10* | Sorting nexin 10 | 7 (001199835) |
|  | PDB5 | 239000 | 8q24 | *TNFRSF11B* | Tumour necrosis factor receptor superfamily, member 11b | 5 (002546) |
|  | CMDR | 218400 | 6q22-23 | *GJA1* | Gap junction protein alpha-1 | 2 (000165) |
|  | PYCD | 265800 | 1q21 | *CTSK* | Cathepsin K | 8 (000396) |
|  | GHDD | 231095 | 7q34 | *TBXAS1* | Thromboxane A synthase 1 | 17 (001061) |
|  | PHOAR1/COA | 259100 | 4q34-35 | *HPGD* | 15-alpha-hydroxyprostaglandin dehydrogenase | 7 (000860) |
|  | LAD3 | 612840 | 11q12 | *FERMT3* | Fermitin family member 3 | 15 (031471) |
|  | LAD3 | 612840 | 11q13 | *RASGRP2* | Ras guanyl nucleotide-releasing protein 2 | 17 (153819) |
|  | SOST1 | 269500 | 17q21 | *SOST* | Sclerostin | 2 (025237) |
|  | RNS | 259775 | 7p22 | *FAM20C* | Family with sequence similarity 20, member C | 10 (020223) |
|  | BOCD | 215045 | 3p21.31 | *PTH1R* | Parathyroid hormone receptor-1 | 16 (000316) |
|  | NHD | 221770 | 19q13.12 | *TYROBP* | TYRO protein tyrosine kinase binding protein | 5 (003332) |
| X-linked OPT | EDA-ID | 300291  300301 | Xq28 | *IKBKG* | Inhibitor of kappa light polypeptide gene enhancer, kinase of, gamma | 10 (001099856) |
|  | OSCS | 300373 | Xq11.1 | *AMER1* | Adenomatous polyposis coli (APC) membrane recruitment protein 1 | 2 (152424) |

^a^Longest protein coding isoform for which all splice junctions are supported by at least one non-suspect RNA (Transcript Support Level (TSL) 1) and which is designated as the principle isoform or a candidate principal isoform (Annotating principal splice isoforms; APPRIS); NCBI RefSeq is in parentheses. OPT: osteopetrosis; BOS: Buschke-Ollendorff syndrome; CMDD: craniometaphyseal dysplasia, autosomal dominant; CAEND: Camurati-Engelmann disease; TDO: trichodentoosseous syndrome; CDD: craniodiaphyseal dysplasia, autosomal dominant; PDB: Paget disease of bone; CMDR: craniometaphyseal dysplasia, autosomal recessive; PYCD: pycnodysostosis; GHDD: Ghosal hematodiaphyseal dysplasia; PHOAR: primary hypertrophic osteoarthropathy, autosomal recessive; COA: cranioosteoarthropathy; LAD3: leukocyte adhesion deficiency type III; SOST1: sclerosteosis 1; RNS: Raine syndrome; BOCD: Blomstrand chondrodysplasia; NHD: Nasu-Hakola disease; EDA-ID: ectodermal dysplasia, with immune deficiency; OSCS: osteopathia striata with cranial sclerosis. Candidate gene list compiled from [Aggarwal 2013; Aker et al. 2012; Bollerslev et al 2013; Sobacchi et al. 2013; Warman et al. 2011].

**ONLINE SUPPLEMENTARY TABLE II.** Candidate genes for proximal renal tubular acidosis (pRTA)

|  | Disorder (Symbol) | OMIM # | Locus | Gene name | Protein | Number of exons (longest isoform)^a^ |
| --- | --- | --- | --- | --- | --- | --- |
| Autosomal recessive  Isolated pRTA | AR pRTA | 604278 | 4q13.3 | *SLC4A4* | Solute carrier family 4 (sodium bicarbonate cotransporter), member 4 | 26 (001098484) |
|  | OPTB3 | 259730 | 8q21.2 | *CA2* | Carbonic anhydrase type 2 | 7 (000067) |
| Syndromic pRTA | FBS | 227810 | 3q26.2 | *SLC2A2* | Solute carrier family 2 (facilitated glucose transporter), member 2 | 11 (000340) |
|  | CTNS | 219800 | 17p13.2 | *CTNS* | Cystinosin | 13 (001031681) |
|  | TYRSN1 | 276700 | 15q25.1 | *FAH* | Fumarylacetoacetase | 14 (000137) |
|  | TYRSN2 | 276600 | 16q22.2 | *TAT* | Tyrosine aminotransferase | 12 (000353) |
|  | TYRSN3 | 609695 | 12q24.31 | *HPD* | 4-hydroxyphenolpyruvate dioxygenase | 14 (002150) |
|  | WND | 277900 | 13q14.3 | *ATP7B* | ATPase, Cu^2+^ transporting, beta polypeptide | 21 (000053) |
|  | Galactosemia | 230400 | 9p13.3 | *GALT* | Galactose-1-phosphate uridylyltransferase | 11 (000155) |
| X-linked | Dent’s disease 1 | 300009 | Xp11.23 | *CLCN5* | Chloride/proton antiporter 5 | 12 (001282163) |
|  | OCRL | 309000 | Xq25 | *OCRL* | Oculocerebrorenal syndrome of Lowe | 24 (000276) |

^a^Longest protein coding isoform for which all splice junctions are supported by at least one non-suspect RNA (Transcript Support Level (TSL) 1) and which is designated as the principle isoform or a candidate principal isoform (Annotating principal splice isoforms; APPRIS); NCBI RefSeq is in parentheses. AR: autosomal recessive; FBS: Fanconi-Bickel syndrome; CTNS: cystinosis; TYRSN: tyrosinemia; WND: Wilson disease; OCRL: oculocerebrorenal syndrome of Lowe. Candidate gene list compiled from [Haque et al. 2012].

**ONLINE SUPPLEMENTARY TABLE III.** Results of ammonium chloride loading test in the proband

| **Biochemical parameter** | **Pre-test** | **Post-test** | **Reference Range** |
| --- | --- | --- | --- |
| **Plasma pH** | 7.32 | 7.26 | (7.35-7.45) |
| **Plasma bicarbonate** | 22.7 mmol/L | 20.7 mmol/L | (22-29 mmol/L) |
| **Plasma partial pressure of carbon dioxide** | 44 mmHg | 46 mmHg | (35-45 mmHg) |
| **Urinary pH** | 5.65 | 5.08 | 4.6-8.0 |

The ammonium chloride loading test measures the ability of the kidneys to excrete acid [Haque et al. 2012].

**ONLINE SUPPLEMENTARY TABLE 4.** ES coverage across osteopetrosis and pRTA candidate genes.

| Gene | HGNC ID | Total coverage | Average coverage | % above 10 reads | % above 20 reads | % above 30 reads |
| --- | --- | --- | --- | --- | --- | --- |
| *AMER1* | 26837 | 277165 | 79.05 | 100 | 100 | 100 |
| *ANKH* | 15492 | 242263 | 120.89 | 88.3 | 85.3 | 84.5 |
| *ATP7B* | 870 | 653266 | 135.59 | 100 | 99.9 | 98.7 |
| *CA2* | 1373 | 185539 | 182.44 | 85.4 | 85.4 | 85.4 |
| *CLCN5* | 2023 | 362656 | 130.64 | 100 | 99.2 | 98.6 |
| *CLCN7* | 2025 | 144748 | 43.38 | 71.9 | 64.1 | 52.1 |
| *CTNS* | 2518 | 182557 | 113.67 | 99.3 | 93.9 | 86.1 |
| *CTSK* | 2536 | 267669 | 201.71 | 100 | 100 | 100 |
| *DLX3* | 2916 | 89722 | 97.1 | 91.3 | 86.5 | 74.4 |
| *FAH* | 3579 | 271480 | 173.91 | 100 | 100 | 100 |
| *FAM20C* | 22140 | 105699 | 54.07 | 58.2 | 48.6 | 45.4 |
| *FERMT3* | 23151 | 100194 | 43.87 | 79.8 | 67.4 | 57.9 |
| *GALT* | 4135 | 273016 | 141.53 | 98 | 93.9 | 92.2 |
| *GJA1* | 4274 | 206037 | 176.25 | 100 | 100 | 100 |
| *HPD* | 5147 | 226432 | 154.88 | 100 | 100 | 100 |
| *HPGD* | 5154 | 166171 | 155.15 | 89.4 | 89.4 | 89.4 |
| *IKBKG* | 5961 | 128682 | 77.33 | 89.6 | 89.6 | 89.3 |
| *LEMD3* | 28887 | 423411 | 141.33 | 83.5 | 80.7 | 76.3 |
| *LRP5* | 6697 | 363113 | 65.78 | 85.8 | 77.6 | 70.8 |
| *OCRL* | 8108 | 436247 | 136.93 | 98.1 | 98.1 | 98.1 |
| *OSTM1* | 21652 | 120214 | 106.86 | 71 | 62.5 | 62.5 |
| *PLEKHM1* | 29017 | 409225 | 119.27 | 100 | 100 | 93.6 |
| *PTH1R* | 9608 | 170919 | 80.13 | 82.9 | 78.2 | 73 |
| *RASGRP2* | 9879 | 183556 | 80.83 | 95.2 | 88.9 | 86.6 |
| *SLC2A2* | 11006 | 310686 | 173.08 | 100 | 100 | 99.3 |
| *SLC4A4* | 11030 | 833683 | 199.78 | 100 | 100 | 99.4 |
| *SNX10* | 14974 | 143696 | 166.51 | 100 | 100 | 99.4 |
| *SOST* | 13771 | 33560 | 49.21 | 35.2 | 35.2 | 35.2 |
| *TAT* | 11573 | 262144 | 165.39 | 100 | 100 | 100 |
| *TBXAS1* | 11609 | 358522 | 163.19 | 95.7 | 94.5 | 85.8 |
| *TCIRG1* | 11647 | 105081 | 35.56 | 62.1 | 55.1 | 47.2 |
| *TGFB1* | 11766 | 95482 | 68.54 | 60.2 | 60.2 | 60.2 |
| *TNFRSF11A* | 11908 | 224805 | 109.61 | 77.7 | 74.6 | 68.7 |
| *TNFRSF11B* | 11909 | 320370 | 234.88 | 92.1 | 92.1 | 92.1 |
| *TNFSF11* | 11926 | 119069 | 112.97 | 79.9 | 77.3 | 77.3 |
| *TYROBP* | 12449 | 71907 | 134.41 | 100 | 100 | 100 |

HGNC ID: Human Genome Organization (HUGO) Gene Nomenclature Committee identification number.

**ONLINE SUPPLEMENTARY REFERENCES**

Aggarwal S. 2013. Skeletal dysplasias with increased bone density: evolution of molecular pathogenesis in the last century. Gene 528:41-45.

Aker M, Rouvinski A, Hashavia S, Ta-Shma A, Shaag A, Zenvirt S, Israel S, Weintraub M, Taraboulos A, Bar-Shavit Z, Elpeleg O. 2012. An SNX10 mutation causes malignant osteopetrosis of infancy. J Med Genet 49:221-226.

Bollerslev J, Henriksen K, Nielsen MF, Brixen K, Van Hul W. 2013. Autosomal dominant osteopetrosis revisited: lessons from recent studies. Eur J Endocrinol 169:R39-57.

Cleiren E, Benichou O, Van Hul E, Gram J, Bollerslev J, Singer FR, Beaverson K, Aledo A, Whyte MP, Yoneyama T, deVernejoul MC, Van Hul W. 2001. Albers-Schonberg disease (autosomal dominant osteopetrosis, type II) results from mutations in the ClCN7 chloride channel gene. Hum Mol Genet 10:2861-2867.

Haque SK, Ariceta G, Batlle D. 2012. Proximal renal tubular acidosis: a not so rare disorder of multiple etiologies. Nephrol Dial Transplant 27:4273-4287.

Lunter G, Goodson M. 2011. Stampy: a statistical algorithm for sensitive and fast mapping of Illumina sequence reads. Genome Res 21:936-939.

Nesbit MA, Hannan FM, Howles SA, Reed AA, Cranston T, Thakker CE, Gregory L, Rimmer AJ, Rust N, Graham U, Morrison PJ, Hunter SJ, Whyte MP, McVean G, Buck D, Thakker RV. 2013. Mutations in AP2S1 cause familial hypocalciuric hypercalcemia type 3. Nat Genet 45:93-97.

Plagnol V, Curtis J, Epstein M, Mok KY, Stebbings E, Grigoriadou S, Wood NW, Hambleton S, Burns SO, Thrasher AJ, Kumararatne D, Doffinger R, Nejentsev S. 2012. A robust model for read count data in exome sequencing experiments and implications for copy number variant calling. Bioinformatics 28:2747-2754.

Rimmer A, Phan H, Mathieson I, Iqbal Z, Twigg SR, Consortium WGS, Wilkie AO, McVean G, Lunter G. 2014. Integrating mapping-, assembly- and haplotype-based approaches for calling variants in clinical sequencing applications. Nat Genet 46:912-918.

Sobacchi C, Schulz A, Coxon FP, Villa A, Helfrich MH. 2013. Osteopetrosis: genetics, treatment and new insights into osteoclast function. Nat Rev Endocrinol 9:522-536.

Taylor JC, Martin HC, Lise S, Broxholme J, Cazier JB, Rimmer A, Kanapin A, Lunter G, Fiddy S, Allan C, Aricescu AR, Attar M, Babbs C, Becq J, Beeson D, Bento C, Bignell P, Blair E, Buckle VJ, Bull K, Cais O, Cario H, Chapel H, Copley RR, Cornall R, Craft J, Dahan K, Davenport EE, Dendrou C, Devuyst O, Fenwick AL, Flint J, Fugger L, Gilbert RD, Goriely A, Green A, Greger IH, Grocock R, Gruszczyk AV, Hastings R, Hatton E, Higgs D, Hill A, Holmes C, Howard M, Hughes L, Humburg P, Johnson D, Karpe F, Kingsbury Z, Kini U, Knight JC, Krohn J, Lamble S, Langman C, Lonie L, Luck J, McCarthy D, McGowan SJ, McMullin MF, Miller KA, Murray L, Nemeth AH, Nesbit MA, Nutt D, Ormondroyd E, Oturai AB, Pagnamenta A, Patel SY, Percy M, Petousi N, Piazza P, Piret SE, Polanco-Echeverry G, Popitsch N, Powrie F, Pugh C, Quek L, Robbins PA, Robson K, Russo A, Sahgal N, van Schouwenburg PA, Schuh A, Silverman E, Simmons A, Sorensen PS, Sweeney E, Taylor J, Thakker RV, Tomlinson I, Trebes A, Twigg SR, Uhlig HH, Vyas P, Vyse T, Wall SA, Watkins H, Whyte MP, Witty L, Wright B, Yau C, Buck D, Humphray S, Ratcliffe PJ, Bell JI, Wilkie AO, Bentley D, Donnelly P, McVean G. 2015. Factors influencing success of clinical genome sequencing across a broad spectrum of disorders. Nat Genet 47:717-726.

Uitterlinden AG, Arp PP, Paeper BW, Charmley P, Proll S, Rivadeneira F, Fang Y, van Meurs JB, Britschgi TB, Latham JA, Schatzman RC, Pols HA, Brunkow ME. 2004. Polymorphisms in the sclerosteosis/van Buchem disease gene (SOST) region are associated with bone-mineral density in elderly whites. Am J Hum Genet 75:1032-1045.

Warman ML, Cormier-Daire V, Hall C, Krakow D, Lachman R, LeMerrer M, Mortier G, Mundlos S, Nishimura G, Rimoin DL, Robertson S, Savarirayan R, Sillence D, Spranger J, Unger S, Zabel B, Superti-Furga A. 2011. Nosology and classification of genetic skeletal disorders: 2010 revision. Am J Med Genet A 155A:943-968.

Williams SE, Reed AA, Galvanovskis J, Antignac C, Goodship T, Karet FE, Kotanko P, Lhotta K, Moriniere V, Williams P, Wong W, Rorsman P, Thakker RV. 2009. Uromodulin mutations causing familial juvenile hyperuricaemic nephropathy lead to protein maturation defects and retention in the endoplasmic reticulum. Hum Mol Genet 18:2963-2974.
